# isoSeQL: comparing long-read isoforms across multiple datasets

**DOI:** 10.1093/bioinformatics/btaf680

**Published:** 2025-12-26

**Authors:** Christine S Liu, Jerold Chun

**Affiliations:** Center for Neurologic Diseases, Sanford Burnham Prebys Medical Discovery Institute, La Jolla, CA 92037, United States; Biomedical Sciences Program, School of Medicine, University of California San Diego, La Jolla, CA 92093, United States; Center for Neurologic Diseases, Sanford Burnham Prebys Medical Discovery Institute, La Jolla, CA 92037, United States

## Abstract

**Motivation:**

Long-read sequencing has made RNA isoform detection and characterization more accessible. While several bioinformatics tools have been developed to examine the data generated by these approaches, a major challenge in the field has been comparing isoform profiles across several samples.

**Results:**

We developed isoSeQL, a tool for compiling long-read transcriptomic data, identifying common and unique isoforms across multiple samples, and extracting and visualizing various metrics. isoSeQL will augment approaches that utilize long-read sequencing to discover novel isoforms and to examine how isoforms vary across different experimental and biological conditions and cell types. We demonstrate how to use isoSeQL with publicly available datasets.

**Availability and implementation:**

isoSeQL is available on Github: https://github.com/christine-liu/isoSeQL and Zenodo:https://doi.org/10.5281/zenodo.15717809.

## 1 Introduction

Advances in long-read sequencing technology have made it possible to examine the transcriptome at isoform resolution. Because short-read RNA-sequencing (RNA-seq) technologies do not capture full-length transcripts, identifying isoforms using short-read RNA-seq requires assembly and inference of exon combinations. In contrast, the long reads obtained through either Oxford Nanopore Technologies (ONT) or PacBio sequencing technologies readily exceed the length of the average mammalian mRNA (∼2–3 kb), making it possible to examine directly the combination of exons that are expressed together in a single transcript (Piovesan *et al.* 2016, Piovesan *et al.* 2019). Isoform diversity has been explored in multiple species and also at the single-cell level (Sharon *et al.* 2013, Gupta *et al.* 2018, Workman *et al.* 2019, Amarasinghe *et al.* 2020, De Paoli-Iseppi *et al.* 2021, Joglekar *et al.* 2021, Leung *et al.* 2021, Palmer *et al.* 2021, Hardwick *et al.* 2022, Veiga *et al.* 2022, Liu *et al.* 2024, Aguzzoli Heberle *et al.* 2025, Vollger *et al.* 2025). Many studies have reported novel isoforms, implicating the existence of a whole set of transcripts that could produce proteins having currently unknown functions, which have not been captured and annotated up to this point. Relatively recent bioinformatics pipelines and tools have been developed to characterize these isoforms and examine their abundance, structure, and protein-coding potential among other properties (Amarasinghe *et al.* 2021).

One such tool is SQANTI3 (Tardaguila *et al.* 2018, Pardo-Palacios *et al.* 2024a). SQANTI3 is widely used for quality control (QC) assessment and annotation of isoforms and has previously been used to evaluate and benchmark other long-read analysis tools (Pardo-Palacios *et al.* 2024b). Isoforms are labeled by gene, structural category, and various other metrics to distinguish novel isoforms from known isoforms and filter out potential artifacts. While SQANTI3 itself does not perform any quantification, it can track read abundances supplied from prior analysis steps. A set of isoforms in a gtf or fasta file format is provided as the input to SQANTI3, and several files classifying the properties of these isoforms are generated as the output.

SQANTI3 is utilized after preceding tools have transformed raw sequencing reads into non-redundant isoforms. Starting from reads, the general workflow involves read QC (tailored to the sequencing technology used), mapping to the genome, generating a unique set of high-quality isoforms, and annotating and filtering with SQANTI3. Downstream analyses can then make use of the SQANTI3 annotations to identify isoform changes between different conditions. For example, SQANTI3 annotations make it possible to look for novel isoforms that may be more highly expressed in disease.

While SQANTI3 has become a standard tool in the field due to its sophisticated QC metrics (Pardo-Palacios *et al.* 2024b), its single-sample design limits its utility for comparative analysis across multiple datasets. The resulting annotated isoforms are labeled with randomized isoform ID numbers (e.g. PB.X.Y) that are not consistent across separately analyzed samples. PB.X.Y in one sample is not necessarily the same isoform as PB.X.Y in another sample, making it difficult to determine whether samples have any isoforms in common. While the recommended workflow suggests pooling samples to create a single transcriptome with consistent isoform naming, this approach does not scale as sample sizes increase.

Several other strategies exist for aggregating isoforms across multiple long-read RNA-seq samples, including gffcompare, cDNA_Cupcake’s chain_samples.py, and TAMA merge (Tseng 2017, Kuo *et al.* 2020, Pertea and Pertea 2020). These tools facilitate transcript model collapsing, and in some cases, quantify isoforms across samples. However, they are not specifically designed for comparative isoform analysis and typically require re-annotating transcript models as a combined set, even if they had already been annotated at the individual sample level. Moreover, these tools are known to be limited to small sample numbers, may result in redundant isoforms (where one sample’s isoform is linked to two isoforms in another sample), or do not track sample characteristics. These limitations highlight the need for a dedicated solution that can integrate and compare SQANTI3 annotations of isoforms and corresponding abundances across samples in an efficient and scalable manner.

isoSeQL is a dedicated tool for aggregating annotated long-read transcriptomes across samples and tracking their associated metadata and abundances. Many different analysis methods, such as IsoQuant (Prjibelski *et al.* 2023), TALON (Wyman *et al.* 2020), and FLAIR (Tang *et al.* 2020), can be used to generate transcript models ahead of annotation with SQANTI3, which is the only requirement for isoSeQL usage. Transcripts from each sample are expected to have undergone QC, mapping, collapsing into non-redundant isoforms, and annotation with SQANTI3. Using SQANTI3 output files from each sample as the input, isoSeQL unifies the isoform IDs, tracks the read counts associated with each isoform from each sample, and returns a consolidated SQLite database of isoforms to which more samples can be added over time. There are two phases to utilizing isoSeQL: (i) generating and populating the database and (ii) querying the database for visualization or downstream analyses. Functions have been written for both phases, outputting an updated database in addition to various tables and plots for visualization.

## 2 Materials and methods

### 2.1 The isoSeQL method

**Figure 1.**
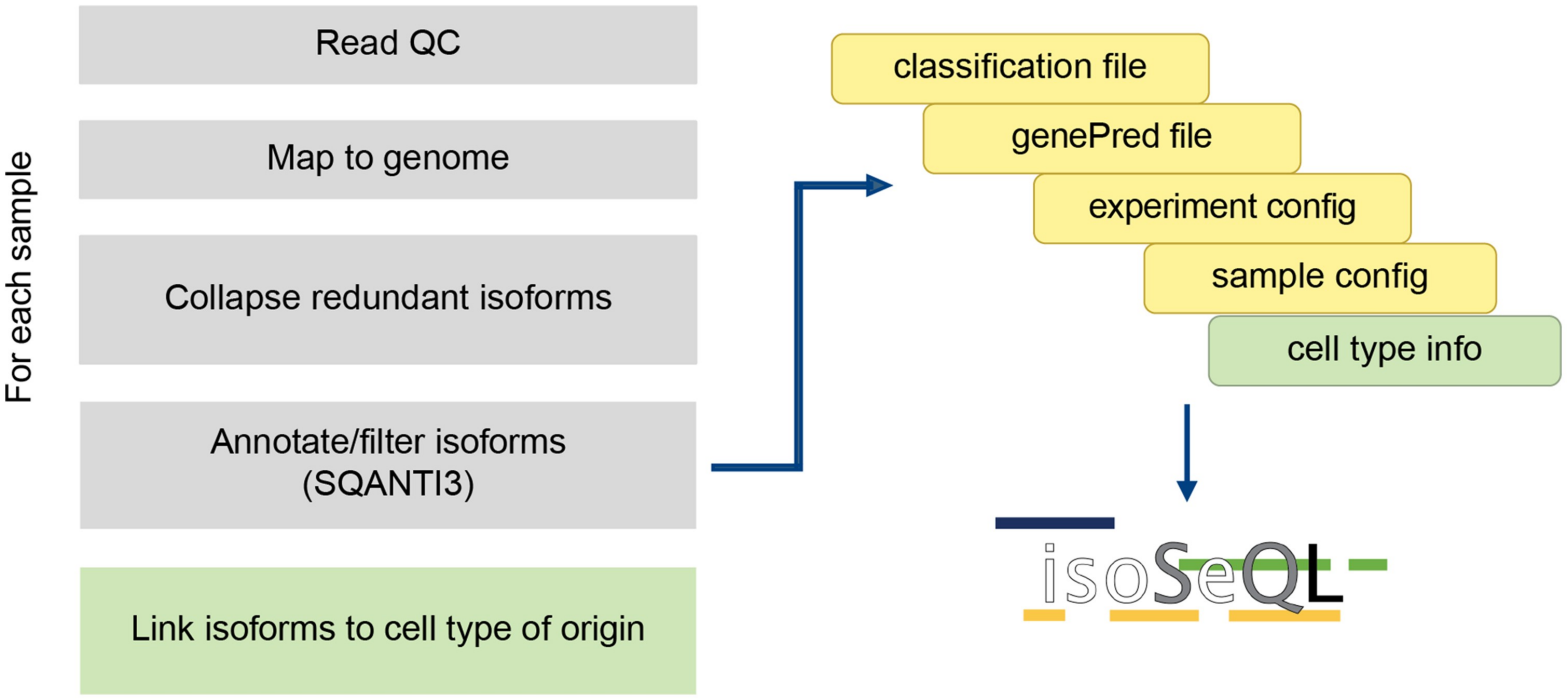
Generalized workflow for processing long-read isoform sequencing data to run through isoSeQL. Every sample is processed individually and added to the database using two SQANTI3 output files and user-supplied config files with sample and experiment information. Additional steps and files for single-cell analysis are indicated in green (“Link isoforms to cell type of origin” and “cell type info”).

isoSeQL consolidates SQANTI3-analyzed samples and provides functions for visualization and quantification (Fig. 1, Tables 1 and 2, available as supplementary data at *Bioinformatics* online). For bulk sequencing analyses, isoSeQL requires two output files from SQANTI3: (i) the classification file listing out annotated isoforms and their corresponding metrics and (ii) the genePred file, which is a tab-delimited file that describes the structure of the transcripts (start/end/exon coordinates). Two user-supplied files with experiment and sample metadata are also used as input to link sample information to the isoforms. Single-cell analysis requires an additional file connecting isoforms to their cellular barcodes and cell type labels. isoSeQL is run in two phases: adding data to the SQLite database and querying the data for downstream analyses.

**Table 1.** Features of gffcompare and isoSeQL.

Tool	Input(s)	Output(s)	Combine isoforms based on intron chain	Track start/end coordinate variability	Track QC metrics and annotation	Track sample information	Functions for visualization
gffcompare	gtf/gff files, reference (optional)	combined gff	✓	x	x	x	x
isoSeQL	classification file, genePred, sample.config, exp.config	isoSeQL database	✓	✓	✓	✓	✓

#### 2.1.1 Populating the database

The first phase of utilizing isoSeQL is adding isoforms to the database. A new SQLite database is initialized, or isoforms can be added to an existing database. The database is populated by first querying each isoform. If it’s already present in the isoform table, then the tables tracking associated read counts are updated. If the isoform is not already present in the table, then it is added along with its associated read counts. For single-cell data, additional tables are populated with associated cellular barcode and cell type information. Separate tables store sample and experiment metadata (Fig. 1, available as supplementary data at *Bioinformatics* online), making it possible to subset and/or filter samples based on various characteristics like sex, age, disease, etc. The use of an SQLite database enables additional samples to be added over time, allowing isoforms from newer experiments to be compared directly to previously observed isoforms.

#### 2.1.2 Isoforms defined by intron chain

isoSeQL defines isoforms by their intron chains. Intron chains are given isoform ID numbers that are used to link entries across different SQLite tables. Intron chains are stored in the isoform table along with SQANTI3 annotations that describe the gene, structural category, novel isoform features, etc. Start and end coordinate combinations linked to the intron chain are stored in a separate table, making it possible to investigate further if these coordinates may correspond to novel transcription start and termination sites.

#### 2.1.3 Functions for querying the database

The second phase of using isoSeQL is querying information from the database. Several functions (Tables 1 and 2, available as supplementary data at *Bioinformatics* online) have been written to extract and/or generate sample metadata, count matrices, plots of isoforms/reads by category, UpSet plots comparing samples, etc These functions do not encompass all the possible ways that one may want to query their data. Users may load the SQLite database in python and write their own custom queries to subset, filter, and plot their data as desired. Example queries are provided on github (http://www.github.com/christine-liu/isoSeQL)

### 2.2 Defining isoforms and example use-cases

#### 2.2.1 GENCODE analysis

We analyzed the GENCODE reference annotation (v39) to quantify how many isoforms can be uniquely defined by their intron chain. The gtf file was downloaded from https://www.gencodegenes.org/human/release_39.html, and a simple analysis was performed to count the number of start/end coordinate combinations present in isoforms with each intron chain. While parsing the gtf file, a python dictionary was populated with (key, value) pairs in which the key was the intron chain, and its corresponding value was a list of the start/end coordinates. We iterated over the keys and counted how many were associated with more than one start/end combination.

#### 2.2.2 Comparison of collapsing strategies

Simulated data were generated to demonstrate different approaches to collapsing transcripts into unique isoforms and how isoSeQL matches isoforms between samples. We selected an isoform (ENST00000440086.1) from *LPAR3* and made various transcripts truncated on either the 3′ or 5′ end while maintaining the same intron chain. Transcripts were collapsed using isoseq collapse (v4.2) to demonstrate how using length cutoffs can affect the resulting isoforms. The transcripts were also analyzed using gffcompare to create a single merged transcriptome that was compared to the isoforms tracked in isoSeQL.

#### 2.2.3 GM12878 bulk example

To demonstrate how isoSeQL can be used to compare isoforms across multiple samples regardless of library preparation or sequencing technology, we downloaded publicly available datasets from long-read RNA-sequencing of the GM12878 cell line. ONT direct RNA (dRNA) and cDNA sequencing data from Workman *et al.* were downloaded from https://github.com/nanopore-wgs-consortium/NA12878. PacBio Iso-Seq of GM12878 by ENCODE was downloaded from https://www.encodeproject.org/experiments/ENCSR962BVU/ (accession code ENCFF913VJX). A recent study utilized PacBio Kinnex to sequence GM12878, and we downloaded their data from SRA (accession SRR29438432). Each sample was processed to remove adapters (as needed), mapped to the GRCh38 primary assembly, collapsed into non-redundant isoforms with isoseq, and annotated/filtered with a modified version of SQANTI3 (Liu *et al.* 2024) using the GENCODE v48 reference annotation. The resulting isoforms were all processed into the same isoSeQL database for consolidation and comparison.

#### 2.2.4 Single-cell example

Two single-cell samples were analyzed with isoSeQL to demonstrate its utility. Processed reads from PacBio Kinnex sequencing of peripheral blood mononuclear cells (PBMCs) were downloaded from https://www.pacb.com/connect/datasets/. The reads for each sample were then mapped to the genome and annotated/filtered with a modified version of SQANTI3 (Liu *et al.* 2024) using the GENCODE v44 reference annotation. Count matrices (gene x cellular barcode) were also downloaded and analyzed with Seurat (v4.3) to identify cell types (Hao *et al.* 2021). The resulting isoforms were processed into an isoSeQL database.

## 3 Results

### 3.1 Unique intron chains in GENCODE

In determining how to define unique isoforms, we analyzed the GENCODE reference to quantify how many isoforms were uniquely described by their intron chain. 244 939 transcripts from the GENCODE v39 annotation were represented by 215 652 unique intron chains. Only 2223 intron chains (1.03%) had more than one transcript with different start/end coordinates associated with it, meaning that over 98% of intron chains represented singular isoforms.

### 3.2 Unique isoform collapse

The main challenge of combining isoform data from multiple samples is determining which isoforms match. RNA degradation, incomplete reverse transcription, and partial sequencing create variability in the start and end coordinates of transcripts, and it’s not a trivial task to distinguish between artifacts and true variation in transcription start and transcription termination sites (Jain *et al.* 2018, Amarasinghe *et al.* 2020). Several different tools upstream of SQANTI3 generate unique isoform sets using various approaches, with many collapsing transcript models that share an intron chain and differ in length on the 5′ and 3′ ends within a specified range (Tseng 2017, Pertea and Pertea 2020). Collapsing isoforms into a unique set is a recommendation of SQANTI3, which is intended to evaluate transcript models and not sequencing reads.

**Figure 2.**
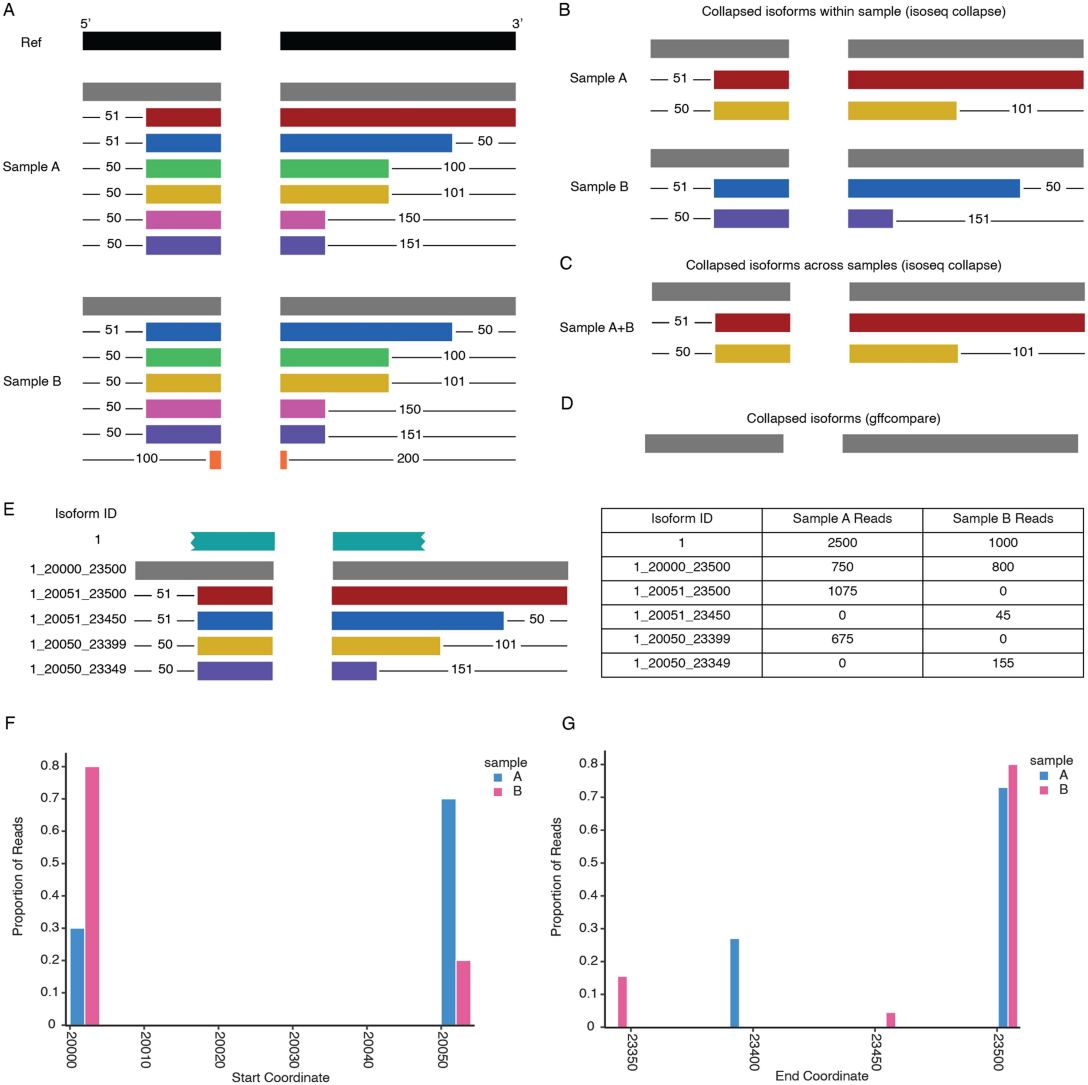
Collapsing redundant isoforms and tracking transcript start/end variability in isoSeQL. (A) Simulated transcripts of LPAR3 for two samples (Sample A and Sample B). Reference exons are shown in black. The numbers indicate the number of base pairs truncated on each end. (B) The result of using isoseq collapse is to remove redundant transcripts within each sample. (C) The result of collapsing isoforms from Sample A with isoforms from Sample B using isoseq collapse. (D) The resulting isoform output from merging isoforms from Sample A with isoforms from Sample B with gffcompare. (E) Schematic of how the isoforms from Sample A and Sample B would be stored in isoSeQL with corresponding read counts. Isoform 1 refers to all isoforms with the same intron chain, while 1_*x*_*y*, where *x* and *y* are the start/end coordinates, refers to isoforms that share the same intron chain but have different start/end coordinates. Coordinates shown are illustrative and not actual genomic coordinates for clarity. (F and G) Start/end site variability plots. Plots showing the proportion of reads supporting the transcript with different start (F) and end (G) coordinates by sample.

We simulated transcripts from *LPAR3*, a two-exon gene, to demonstrate how isoseq collapse and gffcompare collapse isoforms within and across samples (Fig. 2A). Transcripts were truncated on either the 5′ or 3′ end to show how different isoforms were grouped together using isoseq collapse’s cutoffs of 100 base pairs (bp) on the 3′ end and 50 bp on the 5′ end. Collapsing isoforms within each sample (Fig. 2B) showed how the representative isoforms differ slightly between the two samples. Further collapsing the samples together can be achieved by combining all the original transcripts or collapsing the collapsed isoforms together. Both approaches result in the same collapsed isoform set (Fig. 2C). Merging the same isoforms using gffcompare results in only the full-length isoform being reported (Fig. 2D).

Collapsing isoforms across samples by combining all the transcripts together is a workaround for keeping isoform IDs consistent across multiple samples, but it becomes prohibitive as sample sizes increase and may even obscure some sample-to-sample heterogeneity. In preparation for analysis with isoSeQL, each sample is analyzed individually, with isoforms collapsed only within the sample using whichever tool the user deems appropriate for their application. To consolidate isoforms across samples, isoSeQL avoids over-collapsing and the loss of information about start/end variability by not performing further collapsing and instead linking the different start/end coordinate combinations to their intron chain. Each isoform is defined by its intron chain and assigned an isoform ID number. The various start/end combinations are stored separately, making it possible to query these data as needed to identify transcripts that appear to contain the same exons but differ in length at the 5′ and/or 3′ ends. These isoforms have an ID composed of the isoform ID number linked to the intron chain and the start/end coordinates (e.g. *x*_*y*_*z* where *x* is the isoform ID, *y* is the start coordinate, and *z* is the end coordinate). The number of reads associated with a unique intron chain is the sum of reads from all isoforms with that intron chain, regardless of start/end coordinates. Tracking isoforms in this manner allows the flexibility to ignore start/end variability or directly examine the different potential transcription start and termination sites in downstream analyses.

### 3.3 Comparison to gffcompare

gffcompare is a tool designed to merge structurally equivalent transcripts from multiple gff/gtf files, like those produced after generating unique transcript models (Pertea and Pertea 2020). Initially intended to collapse assemblies from different samples, gffcompare is suited for both short-read and long-read isoform studies. Similar to isoSeQL, it identifies matching isoforms based on the intron chain and outputs a merged set of unique isoforms. While gffcompare makes it possible to achieve a unified isoform ID system, isoSeQL has additional features that improve upon simply consolidating isoforms together (Table 1). While gffcompare collapses isoforms into the longest isoform, discarding information about truncated isoforms (Fig. 2D), isoSeQL retains data about shorter isoforms that may result from new transcription start and termination sites. In addition, isoSeQL tracks SQANTI3 QC metrics and annotations, and links sample metadata to the isoforms, creating a comprehensive data object with a set of functions to facilitate downstream analyses (Fig. 2E). For the investigation of variable transcription start and end sites specifically, plots can be generated to display the distribution of reads per sample that start/end at particular coordinates (Fig. 2F and G). Clusters of reads around particular coordinates may indicate a valid start/end to be investigated through orthogonal methods. isoSeQL streamlines the process of comparing isoforms across samples, providing utility beyond the unified isoform ID system achieved by gffcompare.

### 3.4 Bulk RNA-sequencing analysis with isoSeQL

**Figure 3.**
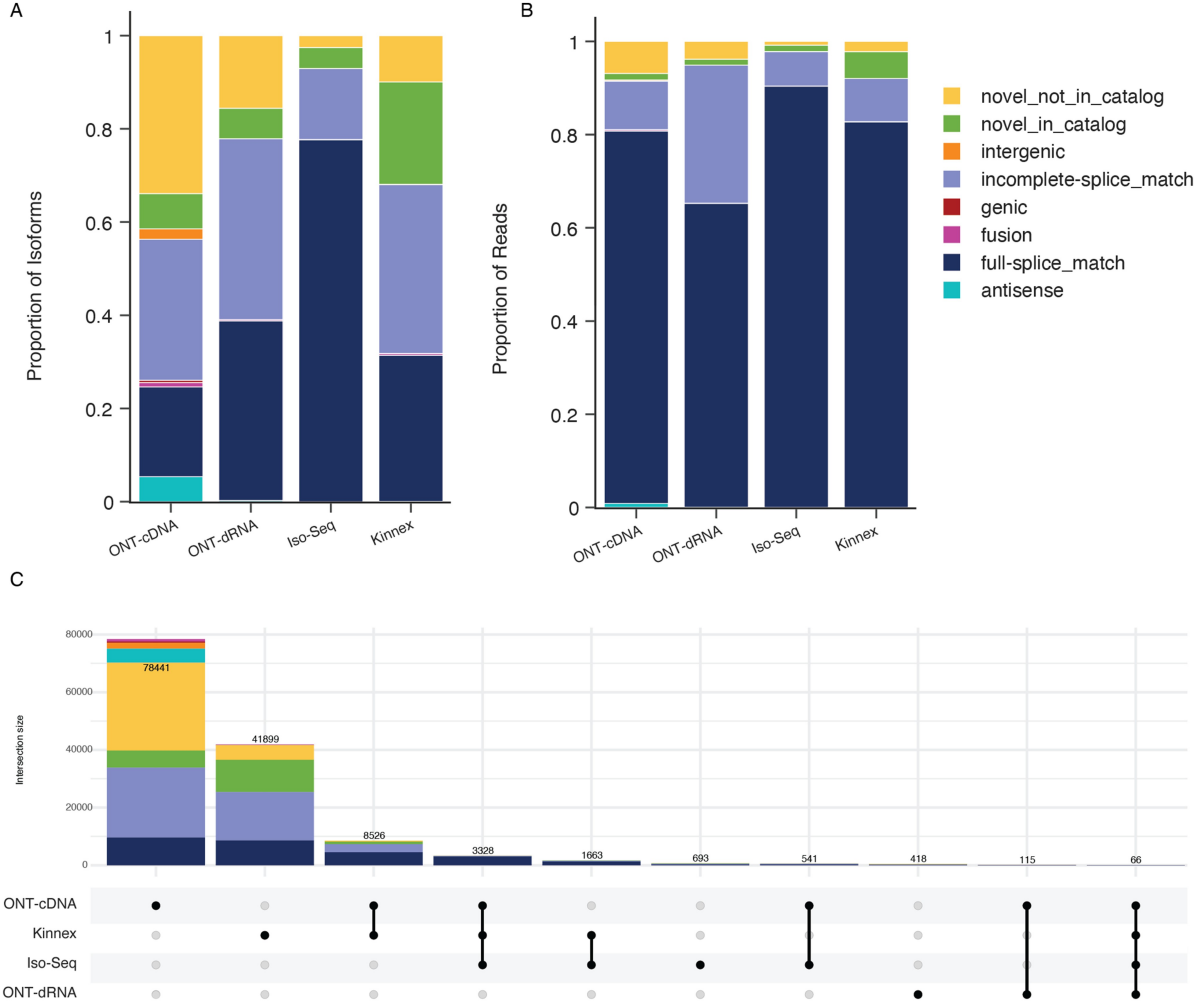
isoSeQL analysis of bulk Iso-Seq data. (A) Plot of proportions of isoforms belonging to different structural categories. (B) Plot showing the proportion of reads from isoforms belonging to different structural categories. (C) UpSet plot showing overlap of isoforms defined by intron chain across samples, colored by structural category. The UpSet plot only shows the top ten intersections.

To demonstrate how isoSeQL can be used to compare various metrics from multiple samples, we downloaded four publicly available datasets that all sequenced the GM12878 cell line, each utilizing a different library preparation or sequencing technology: 1 Iso-Seq sample sequenced on the PacBio Sequel II; 1 bulk Kinnex sample sequenced on the PacBio Revio; and 1 cDNA sample and 1 dRNA sample sequenced with ONT. These data were processed from reads to generate unique sets of high-quality isoforms, and then individually annotated and filtered using SQANTI3. These isoforms were then processed by isoSeQL. Using built-in isoSeQL functions (Table 1, available as supplementary data at *Bioinformatics* online) for querying the data, we were able to examine the relative proportions of isoforms and reads that were assigned to different structural categories (Fig. 3A and B). We saw that even though the same cell line was sequenced for each sample, the number of isoforms in each structural category varied (Fig. 3A). Novel isoforms (novel in catalog, NIC; novel not in catalog, NNC), those not present in the GENCODE v48 reference annotation, made up around 10–40% of the detected isoforms (Fig. 3A). Weighting these isoforms by read count, we saw that a majority of reads across the samples were detected from known isoforms (full-splice match; FSM), and that only a small proportion of reads supported novel isoforms (Fig. 3B). These results fit our expectations that novel isoforms are more rarely expressed. Indeed, 97.2% of novel isoforms were only detected in a single sample (52 957 out of 54 476 novel isoforms). Filtering to identify confident isoforms using normalized counts that account for differences in sequencing depth and the number of samples in which an isoform is detected can easily be performed with isoSeQL. isoSeQL can be used to generate a count matrix from this subset of isoforms that can then be used with downstream tools to examine differential isoform expression and usage. When comparing isoforms detected in each sample, we identified 66 isoforms shared amongst all samples. All 66 isoforms were FSM isoforms, indicating that every novel isoform was found in three or fewer samples (Fig. 3C). This example demonstrates the differences that can be observed from sequencing the same sample with different library preparations, sequencing technologies, and sequencing depths, and showcases isoSeQL’s utility in facilitating such comparisons.

### 3.5 Single-cell isoform data analysis with isoSeQL

**Figure 4.**
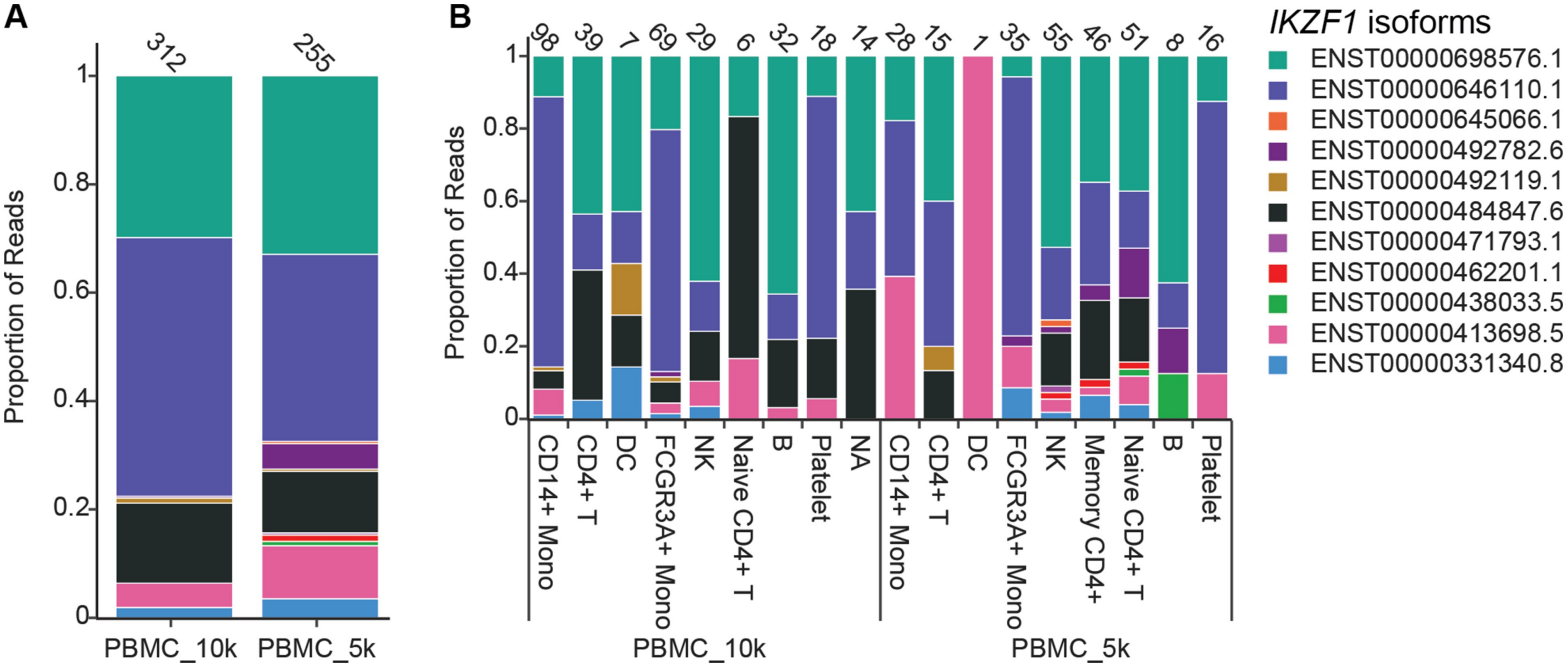
isoSeQL analysis of single-cell Iso-Seq data (A) Read proportion of known isoforms of IKZF1 expressed in each sample, analyzed as a pseudobulk. (B) Read proportion of known IKZF1 isoforms expressed by cell type. Numbers on top of the bars indicate the total read count for that particular cell type.

Single-cell isoform sequencing data can also be processed using isoSeQL (Table 2, available as supplementary data at *Bioinformatics* online). Two publicly available single-cell long-read datasets from peripheral blood mononuclear cells (PBMCs) using the PacBio Kinnex protocol were analyzed using isoSeQL to demonstrate how isoforms from different cell types in separate samples can be compared against each other (PacBio 2023). The only difference between these samples is the number of cells targeted in the library preparation process, which should result in approximately twice as many reads per cell in the sample targeting 5000 cells vs the sample targeting 10 000 cells. Single-cell analysis makes it possible to examine how different cell types are contributing to overall gene and isoform expression. Starting by examining the data as a pseudobulk and then splitting by cell type, we observed that generally the proportions of reads and isoforms from each structural category remained constant across the different cell types (Fig. 2, available as supplementary data at *Bioinformatics* online). No cell type displayed elevated expression of novel isoforms relative to the others. On the gene level, we identified several genes with more than one known isoform contributing to overall expression. For many, the major isoform (highest proportion of reads) did not change between cell types; however, some genes had varying isoform expression profiles across different cell types. *IKZF1* is an example of one of those genes. Pseudobulk analysis indicated that ENST00000698576.1 and ENST00000646110.1 were the highest expressed isoforms (Fig. 4A). Splitting the reads by cell type of origin showed major isoform switching between different cell types (Fig. 4B). Major isoform switching is the case in which the highest expressed isoform in different conditions is not the same. ENST00000646110.1 was the major isoform in three cell types (CD14+ monocytes, FCGR3A+ monocytes, and platelets), while ENST00000698576.1 was the major isoform in CD4+ T cells, natural killer (NK) cells, and B cells. Observing major isoform switching between cell types, such as this, could be indicative of genes having varying functions and roles in different cell types.

## 4 Conclusion

This report describes isoSeQL, a program for comparing full-length RNA isoform profiles resulting from SQANTI3 analysis. isoSeQL was designed specifically for the comparative analysis of isoforms that are not addressed through other bioinformatics packages and can readily be added into current workflows. As the field progresses and improves upon existing technology and tools, new versions of the tools for each step preceding isoSeQL and SQANTI3 can be easily swapped out. This enables the user to tailor the QC, mapping, and collapsing steps to the technology that was used (i.e. different tools are more suited for PacBio vs ONT outputs). SQANTI3 can use the gtf outputs from tools like FLAIR, IsoQuant, and TALON so that an analysis pipeline can utilize the mapping, correction, and transcript model building of many tools in combination with the detailed characterization and filtering by SQANTI3 prior to aggregation using isoSeQL. With this modularity, isoSeQL has many potential use cases in facilitating comparisons between different transcript model tools, sequencing library preparations, sequencing technologies, diseases, cell types, etc Looking forward, isoSeQL has the potential to support atlas-scale isoform annotation efforts by combining a dynamic database that can be updated continuously with flexible metadata integration.

Future iterations of isoSeQL will include improvements to the current implementation. Currently, isoSeQL only tracks long-read abundances (i.e. read counts); however, matching short-read sequencing data can be mapped to the long-read-generated transcriptome for junction confirmation and higher-depth estimates of relative abundance. Incorporating short-read counts will not only provide further validation of novel junctions but will also allow correlations between short- and long-read expression data. Additionally, more functions can be written to generate plots and tables for queries that are not currently included. In summary, the isoSeQL package enables comparative isoform analysis across diseases, cell types, and a variety of other experimental conditions while being designed for broad compatibility with various upstream tools.

## Supplementary Material

btaf680_Supplementary_Data

## Data Availability

Example bulk sequencing datasets were downloaded from https://github.com/nanopore-wgs-consortium/NA12878 (ONT dRNA and cDNA), https://www.encodeproject.org/experiments/ENCSR962BVU/ (accession code ENCFF913VJX; PacBio Iso-seq), and the NCBI Sequence Read Archive (accession SRR29438432; PacBio Kinnex). These datasets were accessed in June 2025. Example single-cell datasets were made publicly available through PacBio (https://downloads.pacbcloud.com/public/dataset/Kinnex-single-cell-RNA/). DATA-SQ2-PBMC_10kcells and DATA-SQ2-PBMC_5kcells were accessed in November 2023. The modified version of SQANTI3 can be found on github: github.com/christine-liu/SQANTI3/tree/SQANTICL (Liu *et al.* 2024). isoSeQL can be found on github: https://github.com/christine-liu/isoSeQL and Zenodo: https://doi.org/10.5281/zenodo.15717809.
