# Supplementary material for: isoSeQL: comparing long-read isoforms across multiple datasets
Source: Bioinformatics. 2025 Dec 26;42(1):btaf680. doi: 10.1093/bioinformatics/btaf680 (PMC12790818; doi:10.1093/bioinformatics/btaf680)
Supplement: btaf680_Supplementary_Data [file btaf680_supplementary_data.zip › Liu_isoSeQL_supplementary.pdf]

Supplementary Material for

isoSeQL: comparing long-read isoforms across multiple datasets

Christine S. Liu[1,2,*], Jerold Chun[1]

[1]Sanford Burnham Prebys Medical Discovery Institute, La Jolla, CA 92037

[2]Biomedical Sciences Program, School of Medicine, University of California San Diego, La Jolla, CA 92093

[*]Corresponding author, csl022@ucsd.edu

This PDF file includes:

- Supplementary Tables 1 to 2
- Supplementary Figures 1 to 2

Supplementary Table 1. Commands for built-in queries to generate tables and plots from bulk/pseudobulk data

Command	Output(s)
**expInfo**	Table of all samples/experiments currently in database and associated info
**genCounts**	Table with the number of genes, isoforms defined by intron chains, isoforms with start/end coordinates, novel isoforms, and reads per experiment
**summary**	1. Table with all isoforms defined by intron chains and corresponding information (gene, category, etc) corresponding read counts per experiment<br>2. Table with all isoform information (including start/end coordinates) and corresponding read counts per experiment
**geneTx**	Table of number of isoforms per gene
**isoProp**	1. Table and stacked bar plot of proportion of isoforms that belong to each structural category<br>2. Table and stacked bar plot of proportion of reads supporting isoforms that belong to each structural category
**bed**	bed file per experiment with isoforms colored by structural category to load into the UCSC Genome Browser or Integrative Genomics Viewer
**IEJtab**	Table of isoforms with intra-exonic junctions (IEJs) and corresponding read counts per experiment
**countMatrix**	Matrix of read counts per isoform or gene per experiment, intended for use with tappAS for differential isoform expression analysis
**tappASgff**	tappAS-compatible gff of isoforms
**upset**	UpSet plot showing the intersection of isoforms in each experiment colored by structural category
**FSM_count**	List of genes and corresponding number of FSM isoforms
**FSM**	Stacked bar plot of relative read count for each known isoform of a gene per experiment
**varEnds_count**	List of isoforms defined by intron chains and the number of different start/end coordinates associated with it
**varEnds**	Plots showing the distribution of start and end coordinates for each isoform for each experiment

Supplementary Table 2. Commands for built-in queries to generate tables and plots from single-cell data

Command	Output(s)
**SCsummary**	1. Table with all isoforms defined by intron chains and corresponding information (gene, category, etc) and read counts per cell type per experiment<br>2. Table with all isoform information (including start/end coordinates) and corresponding number of cells that expressed each isoform
**SCisoProp**	1. Stacked bar plot of proportion of isoforms that belong to each structural category by cell type per experiment, normalized to total number of isoforms in the experiment<br>2. Stacked bar plot of proportion of isoforms in each structural category by cell type per experiment, normalized to number of isoforms per cell type<br>3. Stacked bar plot of proportion of reads supporting isoforms that belong to each structural category by cell type per experiment, normalized to total number of isoforms in the experiment<br>4. Stacked bar plot of proportion of reads supporting isoforms in each structural category by cell type per experiment, normalized to number of reads per cell type
**SCFSM**	Stacked bar plot of relative read count for each known isoform of a gene by cell type per experiment
**SCcountMatrix**	Matrix of read counts per isoform or gene per cell type per experiment
**SCIEJ**	Table of isoforms with (IEJs) and corresponding read counts per cell type per experiment

Supplementary Figures

isoform
id (INT)
chr (TEXT)
strand (TEXT)
junctions (TEXT)
gene (TEXT)
iso_exons (INT)
subcategory (TEXT)
canonical (TEXT)
IEJ (TEXT)
category (TEXT)

isoform_ends
id (TEXT)
isoform_id (INT)
chr (TEXT)
start (INT)
end (INT)
ex_sizes (TEXT)
ex_starts (TEXT)

counts
isoform_id (INT)
exp (INT)
read_count (INT)

ends_counts
ends_id (TEXT)
exp (INT)
read_count (INT)

exp
id (INT)
sample_id (INT)
RIN (REAL)
seq_date (DATE)
platform (TEXT)
method (TEXT)
vMap (TEXT)
vReference (TEXT)
vAnnot (TEXT)
vLima (TEXT)
vCCS (TEXT)
vIsoSeq3 (TEXT)
vCupcake (TEXT)
vSQANTI (TEXT)
exp_name

sampleData
id (INT)
sample_name (TEXT)
tissue (TEXT)
disease (TEXT)
age (INT)
sex (TEXT)

versionInfo
name (TEXT)
visoSeQL (TEXT)

PBID
PBID (TEXT)
exp (INT)
ends_id (TEXT)

txID
tx (TEXT)
exp (INT)
isoform_id (INT)
gene (TEXT)
ends_id (TEXT)

scInfo
id (INT)
exp (INT)
barcode (TEXT)
celltype (TEXT)

scCounts
isoform_id (INT)
scID (INT)
read_count (INT)

scCounts_ends
ends_id (TEXT)
scID (INT)
read_count (INT)

Supplementary Figure 1. isoSeQL SQLite database tables. Table schematic to show what types of information are stored in each table. Keys used to link table entries are in matching colors.

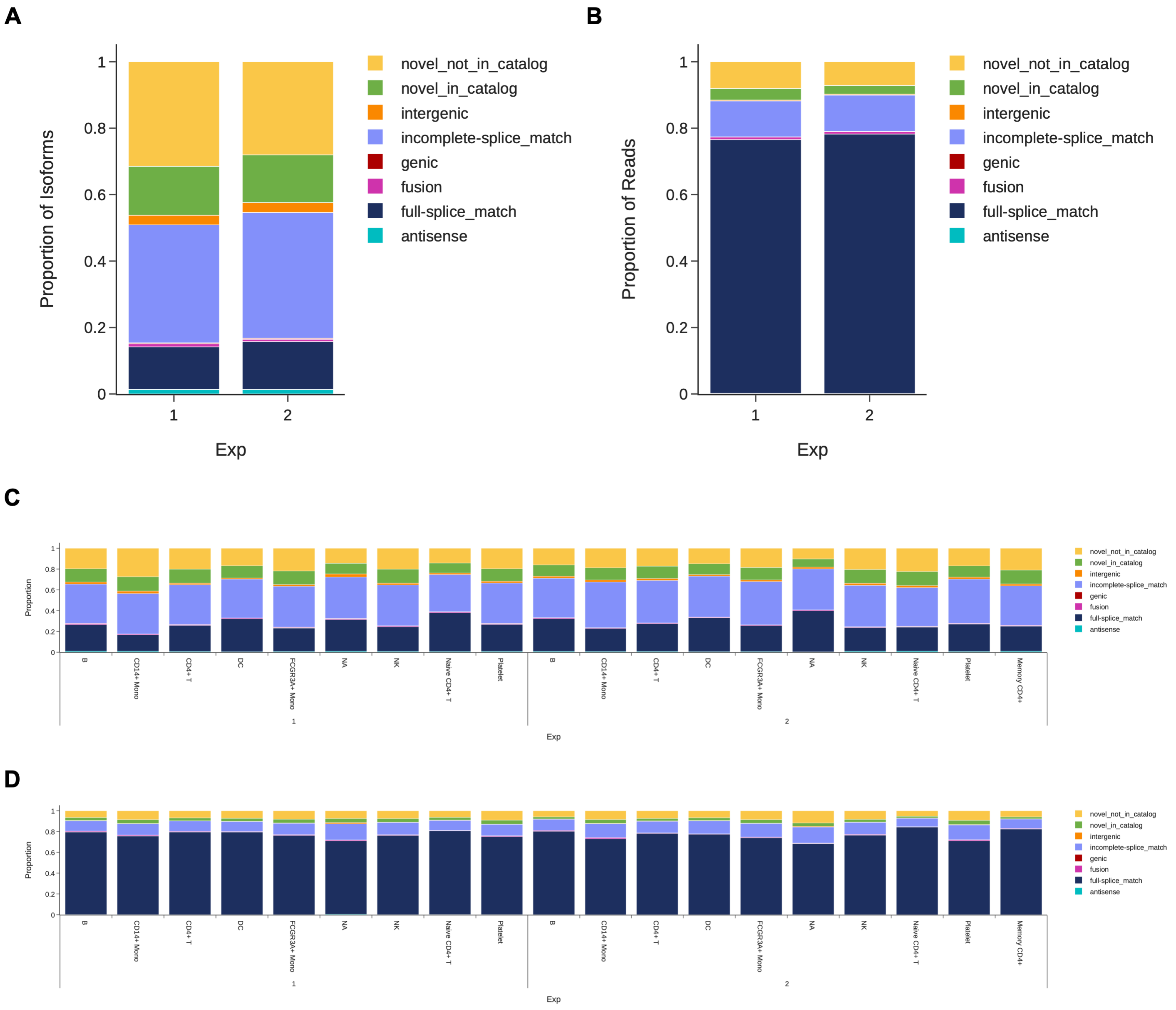

Supplementary Figure 2. Single-cell isoform structural breakdown. *(A-B)* Isoform (A) and read (B) structural category proportions when treating each sample as a pseudobulk. *(C-D)* Normalized structural category isoform (C) and read (D) proportions per cell type in each sample. Exp 1 refers to the PBMC sample targeting 10000 cells; Exp 2 refers to the PBMC sample targeting 5000 cells.
